# 1Design of the **P**rimary P**re**vention **Par**ameters **E**valuation (**PREPARE**) trial of implantablecardioverter defibrillators to reduce patient morbidity [NCT00279279]

**DOI:** 10.1186/1745-6215-7-18

**Published:** 2006-05-25

**Authors:** Bruce L Wilkoff, Richard Stern, Brian Williamson, Mark Wathen, Keith Holloman, Ann Fieberg, Mark Brown

**Affiliations:** 1 The Cleveland Clinic Lerner College of Medicine, Case Western Reserve University, Cleveland, OH, USA; 2 Doctors Medical Center, San Pablo, CA, USA; 3 William Beaumont Hospital, Troy, MI, USA; 4 Vanderbilt University, Nashville, TN, USA; 5 Medtronic, Inc, Minneapolis, MN, USA

## Abstract

**Background:**

Implantable Cardioverter Defibrillator (ICD) therapy has been proven to be beneficial and efficacious for the treatment of serious ventricular tachyarrhythmias in primary prevention patients. However, primary prevention patients appear to have a lower incidence of ventricular arrhythmias in comparison to secondary prevention patients and consequently likely experience a higher proportion of detections due to supraventricular arrhythmias. Recent trials have demonstrated that strategic and specific programming choices reduce the number of inappropriate shocks and that anti-tachycardia pacing (ATP) is an effective alternative to shock therapy for many sustained ventricular arrhythmias.

**Methods:**

The **P**rimary P**re**vention **Par**ameters **E**valuation (PREPARE) study is a multi-center cohort study, evaluating the efficacy of a pre-specified strategic profile of VT/VF detection and therapy settings in 700 primary prevention patients in an effort to safely reduce the number of shock therapies delivered. The patients, both with and without cardiac resynchronization therapy, are compared to a well-qualified set (n = 691) of historical controls derived from the MIRACLE ICD and EMPIRIC trials. This manuscript describes the design of the PREPARE study. The study results, to be presented separately, will characterize the efficacy of this programming set (PREPARE) compared with physician-tailored programming (MIRACLE ICD and EMPIRIC).

## Background

Implantable defibrillator therapy terminates ventricular tachyarrhythmias and improves survival of patients in multiple populations at risk of sudden cardiac death. [1-3] Initially, ICD therapy was applied only to secondary prevention patients: that is, those who had been resuscitated from a cardiac arrest or other sustained ventricular tachyarrhythmias. The impact of other studies, such as MADIT, MUSTT, MADIT II and SCD-HeFT, as well as the CMS national coverage decision in January of 2005, is that the large majority of patients currently receiving ICDs have primary prevention indications and no symptomatic arrhythmias prior to ICD implantation. [4-8]

There are substantial differences in the clinical and rhythm characteristics of patients with primary and secondary prevention indications for ICD therapy. However, it is an important goal to minimize the morbidity of the therapy for all ICD patients. Patients who receive multiple shocks have difficulty adjusting to the ICD implantation, due to increased anxiety and depression. [9,10] In addition, patients who have not had a significant life-threatening arrhythmic experience such as sudden cardiac death may be less accepting of unnecessary shock therapies.

The EMPIRIC and Painfree Rx studies have shown that a standardized set of VT/VF parameters extensively utilizing SVT discriminators and anti-tachycardia pacing (ATP) therapies can be effective in reducing patient shocks after ICD implantation for a mixed population of primary and secondary prevention patients. [11,12] It is the purpose of the PREPARE trial to evaluate strategies specifically designed to reduce shocks in a population of patients with primary prevention ICD indications with and without cardiac resynchronization indications and therapy.

## Methods/Design

### Hypothesis

The PREPARE study examines the hypothesis that a pre-specified strategic profile of tachyarrhythmia detection and therapy parameters is able to reduce the overall morbidity of ICD therapy in patients with primary prevention ICD indications with or without cardiac resynchronization therapy.

### Primary endpoint

Shock related morbidity is measured by the Morbidity Index and expressed through the incidence density. However, the reduction of shocked episodes in primary prevention patients via the use of a specific programming profile is useful only if it does not result in an increase in arrhythmogenic syncope and untreated sustained symptomatic VT/VF events. The Morbidity Index measures the benefit of reducing the number of shocks while also accounting for the possible side effects of 1) treating only faster tachyarrhythmias (cycle-lengths greater than 330 ms) and 2) using a longer programmed delay before treating ventricular tachyarrhythmias with defibrillation.

#### Morbidity index

The components of the Morbidity Index are:

• Syncope (secondary to tachyarrhythmia or presumed tachyarrhythmia)

• Untreated sustained symptomatic VT/VF events

• Episodes of spontaneous ventricular tachyarrhythmia that result in device-delivered shock

• Episodes that result in an inappropriate device-delivered shock.

#### Incidence Density (ID)

The Morbidity Index incidence density is calculated by dividing the total number of Morbidity Index events by the total number of years of follow-up. The primary endpoint is the difference in Morbidity Index incidence density between the study patients programmed to the PREPARE settings and the control population programmed to physician-tailored programming.

### Secondary endpoint

#### Morbidity Tachycardia Index

The key secondary endpoint is the difference in Morbidity Tachycardia Index incidence density, which adds anti-tachycardia pacing events to the Morbidity Index as a reflection of all tachycardia events, between patients programmed to the PREPARE settings and the control population.

### Design

The PREPARE study is a prospective, single arm, multi-center cohort study designed to evaluate the efficacy of a pre-specified strategic profile of VT and VF detection and therapy settings designed for ICD indicated patients with no history of spontaneous sustained symptomatic VT or VF. All study patients received a Medtronic Marquis family ICD system with or without capacity for cardiac resynchronization implanted between October 2003 and April 2005. Approximately 700 patients were enrolled in the United States and the Netherlands and were programmed to the PREPARE parameters and followed for endpoints for 12 months after PREPARE programming.

For the purposes of this study, primary prevention patients are defined as those patients indicated for ICD or CRT + ICD implantation without a baseline history of ventricular fibrillation, ventricular flutter, monomorphic VT, polymorphic VT, or Torsades de Pointes. Patients may have a history of cardiovascular syncope, since the PREPARE findings are intended to be generalized to the primary prevention patient population seen in clinical practice.

• Inclusion criteria required:

○ Initial implantation for primary prevention ICD indications or

○ Prior ICD implantation within 6 months without subsequent

spontaneous VT/VF episodes

• Patients were excluded for:

○ History of spontaneous sustained symptomatic ventricular arrhythmias,

○ An electrophysiology test in the past, with sustained inducible VT < 180 bpm,

○ Any ICD implanted greater than 6 months prior to the study,

○ An ICD implanted within the previous 6 months, with subsequent history of a spontaneous episode of VT or VF appropriately treated with either ATP or shock.

○ Heart transplant

○ Mechanical right heart valve.

### Control population

The control population consists of the prospectively collected shock, tachycardia and event data combined from two randomized clinical ICD trials; the MIRACLE ICD Trial[13] and the EMPIRIC Trial. [11] Both of these trials included both primary and secondary prevention patients, but only the primary prevention patients were included in the control population for the PREPARE analysis.

Originally, the control population included only the primary prevention patient data from the MIRACLE ICD Trial. The MIRACLE ICD Trial was a multi-center randomized trial of 978 CRT-ICD patients, designed to assess the safety and effectiveness of biventricular CRT in primary and secondary prevention patients. However, in contrast to the MIRACLE ICD Trial which enrolled only patients receiving a cardiac resynchronization defibrillator (CRT+ICD), the PREPARE Study includes primary prevention patients implanted with either an ICD or CRT+ICD device. After an interim review of baseline characteristics during the enrollment phase of the PREPARE Trial, it became evident that 65% of the patient population did not require a cardiac resynchronization device. Therefore, the PREPARE Trial population was comprised of patients with more favorable clinical characteristics than the advanced heart failure patients enrolled in the MIRACLE ICD Trial. This imbalance threatened to falsely skew the results of the study, making it difficult to discern whether the potential efficacy of the PREPARE parameters would be the result of the recruitment of patients into the PREPARE study with less severe disease.

To address the imbalanced baseline characteristics between the PREPARE patients and the original control population, the primary prevention patients from the physician-tailored arm of the EMPIRIC study were combined with MIRACLE ICD control patients to produce the control population.

The EMPIRIC study was a multi-center randomized trial of 900 ICD patients, designed to compare standardized vs. physician-tailored VT/VF programming in both primary and secondary prevention patients. [11] The study was recently completed, and its patient population is thought to better reflect the higher ratio of ICD implant indications seen during enrollment into the PREPARE study than the MIRACLE ICD Trial, reported in 2003. [13] Additionally, there were a large number of patients (276) randomized in the EMPIRIC Trial to the physician-tailored arm who met the PREPARE Trial definition of primary prevention. These patients, along with the 415 primary prevention patients from the MIRACLE ICD Trial, have been combined, producing a total of 691 patients in the historical control sample.

When compared with PREPARE patient baseline demographics, the patient characteristics of the combined physician-tailored arm are not clinically different. The baseline clinical data for the study and control patients is listed in Table 1. This table includes baseline demographics which were collected in PREPARE, MIRACLE ICD and EMPIRIC. Demographics for MIRACLE ICD and EMPIRIC are shown separately and in combination.

**Table 1:** Patient Demographics

	**PREPARE Patients** ** (N = 700)**	**MIRACLE ICD Patients** **(N = 415)**	**EMPIRIC Patients** **(N = 276)**	**Combined Control (MIRACLE ICD and EMPIRIC) Patients** **(N = 691)**
**Gender (N, %)**				
Male	555 (79.3%)	310 (74.7%)	222 (80.4%)	532 (77%)
Female	145 (20.7%)	105 (25.3%)	54 (19.6%)	159 (23%)

**Age (years)**				
Mean ± Standard Deviation	67.4 ± 12.2	65.4 ± 11.5	65.6 ± 12	65.5 ± 11.7
Median	68.7	67.9	67.3	67.7
Minimum – Maximum	19.2 – 92.2	31 – 89	23.3 – 91.4	23.3 – 91.4
n (%)	700 (100%)	415 (100%)	276 (100%)	691 (100%)

**Baseline Left Ventricular Ejection Fraction (%)**				
Mean ± Standard Deviation	27.7 ± 10.5	21 ± 6.8	30.3 ± 11	24.7 ± 9.8
Median	25	20	30	25
Minimum – Maximum	5 – 80	6 – 35	5 – 70	5 – 70
n (%)	691 (98.7%)	413 (99.5%)	273 (98.9%)	686 (99.3%)

**Baseline New York Heart Association Classification (N, %)**				
Class I	117 (16.7%)	0 (0%)	36 (13%)	36 (5.2%)
Class II	297 (42.4%)	137 (33%)	102 (37%)	239 (34.6%)
Class III	268 (38.3%)	237 (57.1%)	36 (13%)	273 (39.5%)
Class IV	18 (2.6%)	41 (9.9%)	3 (1.1%)	44 (6.4%)
Not Collected*	0 (0%)	0 (0%)	99 (35.9%)	99 (14.3%)

**Ischemic Cardiomyopathy**	488 (69.7%)	238 (57.5%)	155 (56.2%)	393 (57.0%)

**Myocardial Infarction (N, %)**	421 (60.1%)	193 (46.6%)	192 (69.6%)	385 (55.8%)

N/A = not collected

*NYHA Class was only collected in the EMPIRIC Trial for patients with documented heart failure

#### Programming

The strategies for reducing shock morbidity are to 1) reduce over-treatment of self-terminating tachycardias, 2) reduce the mis-identification of supraventricular arrhythmias as ventricular arrhythmias, and 3) terminate ventricular tachycardia with anti-tachycardia pacing as frequently as possible. Since the patients enrolled in this trial had never experienced a sustained ventricular arrhythmia, the parameters were chosen to not treat: 1) slow and presumably less symptomatic tachycardias, and 2) faster but self-terminating arrhythmias.

The exact tachycardia detection and therapy parameters for the PREPARE study are listed in Table 2. In summary two therapy zones are programmed, treating all rhythms of cycle length less than 330 ms that meet the duration criteria of 30 of 40 intervals. A single anti-tachycardia burst is provided for tachycardias between 330 and 240 ms. If the rhythm persists after ATP, a shock is provided. Rhythms faster than 240 ms receive shocks as the first therapy. A monitor zone is provided for tachycardia between 360 and 330 ms. Many SVT discriminators are programmed ON.

**Table 2:** PREPARE Study VT/VF Programming

**ICD Parameter**	**Marquis/Maximo Family Programmed Value**
**INDUCTION**	

VF Detection	ON
VFDI	330 ms
VF NID	30/40
VF RNID	9/12

**DUAL CHAMBER**	

AF/AFl	ON
Sinus Tachcardia	ON
1:1 VT-ST Boundary	66%
Other 1:1 SVT	OFF
SVT Limit	300
Stability	OFF

**SINGLE CHAMBER**	

Wavelet	On, Match Threshold 70%
Stability	40
Onset	88%

**ALL**	

VF Detection	ON
VFDI	330 ms
VF NID	30/40
VF RNID	9/12
FVT	Via VF
FVTDI	240 ms
VT Detection	Monitor
VTDI	360 ms
VT NID	32
HR Timeout	OFF
VF Rx 1–6 Status	ON
VF Rx 1–6 Energy	30 or 35J maximum output of the ICD
VF Rx 1–4 Pathway	AX->B
VF Rx 5–6 Pathway	B->AX
VF Confirmation	YES
FVT Rx 1–6 Status	ON
FVT Rx 1 Type	Burst
FVT Rx 1 Initial # Pulses	8
FVT Rx 1 R-S1 Interval	88%
FVT Rx 1 # sequence	1
FVT Rx 1 Smart Mode	OFF
FVT Rx 2–6 Type	CV
FVT Rx 2–6 Energy	30 to 35J maximum output of ICD
FVT Rx 2–5 Pathway	AX->B
FVT Rx 6 Pathway	B->AX
VT Rx 1–6	OFF
Other Diagnostic and Therapy parameters	Nominals

The tachycardia detection and therapy parameters for the MIRACLE ICD and EMPIRIC patients included in the control arm were chosen at the discretion of the investigators and are referred to as "physician-tailored" parameters. [14]

#### Data collection

These data are collected at the 6 and 12 month scheduled visits and at unscheduled visits: cardiovascular adverse events, cardiovascular medications, VT/VF and SVT episodes, and patient diary.

Adverse events of syncope, near-syncope, and dizziness are collected between visits in a patient diary and evaluated for arrhythmogenic syncope, i.e. syncope or near syncope caused by a VT/VF or SVT episode, and symptomatic VT/VF events i.e. VT/VF events that result in the symptoms of near syncope or dizziness. All events in the PREPARE, EMPIRIC and MIRACLE ICD Trials will be adjudicated by the PREPARE Adverse Event Advisory Committee (AEAC) to identify arrhythmogenic syncope, near-syncope, and dizziness. The review includes the details surrounding each adverse event and the available EGM data for episodes occurring within 24 hours of the event. For the historical controls, where these adverse events were not as rigorously collected, the event will be adjudicated when the record indicated syncope, near-syncope, or dizziness in the narrative of an adverse event.

The differences in data collection methods between studies are always of concern when utilizing a historical control. There are two particular sources of bias when comparing the primary and key secondary endpoints in the PREPARE study to the historical control data: 1) adverse event collection and 2) incomplete adjudication of VT/VF episodes.

Patients in the PREPARE study are required to record adverse events of syncope, near-syncope, and dizziness in a patient diary, in an effort to increase the probability of collecting all occurrences of these events. By contrast, the MIRACLE ICD study relied on patient recall at study visits, and only system and procedure-related adverse events were collected in the EMPIRIC study. These methods of collection in the historical control group likely led to underreporting of syncope related events, and therefore decreased the probability of arrhythmogenic syncopal or symptomatic VT/VF event discovery during these studies. The expected consequence of this data limitation is that higher rates of arrhythmogenic syncope and symptomatic VT/VF events will be recorded in the PREPARE study.

Not all episodes in the MIRACLE ICD study were originally adjudicated for the spontaneous/induced and true VT/true SVT classifications. To avoid the bias that would be incurred by dropping these episodes with missing information, these will be reviewed and classified by an internal Medtronic VT/VF scientist prior to the completion of the PREPARE study.

## Discussion

### PREPARE programming strategies

#### 1) Strategies to reduce shocks for VT/VF

##### ○ longer detection duration

VF initial beats to detect is set to 30 of 40 beats. At least 25% of ICD-detected VF is non-sustained VT/VF[15-17] The extended detection duration may decrease unnecessary shocks by allowing for spontaneous termination of non-sustained VT/VF that would otherwise be treated with nominal settings of shorter detection duration.

##### ○ ATP for FVTs 330–240 ms

One sequence of ATP will be delivered for FVTs using the FVT via VF zone. Approximately 81% of ICD-detected VF (<320 ms) is monomorphic VT. Monomorphic VT can be pace terminated 75% of the time with one sequence of ATP. [12,15,18,19] It is anticipated that programming a single sequence of ATP will reduce the use of shock therapy in this patient population, and thereby improve the patients' quality of life.

##### ○ maximum output for all VF therapy sequences and FVT sequence 2–6

An initial high output shock may improve first shock success and thus reduce the likelihood of multiple shock episodes. In addition, the slightly longer charge time may allow more non-sustained VT/VF episodes to terminate before the first shock.

#### 2) Strategies to reduce shocks for SVTs

##### ○ tachycardia detection at 330 ms

A treated cut-off of 320 ms has been used in several large primary prevention patient populations. [7,14,20] In the latter two studies, investigators recommended a treatment cut-off between 330 and 350 ms. Detection of only tachyarrhythmias faster than 330 ms should reduce the inappropriate detection of SVTs.

##### ○ VT monitor zone for slow VTs

One recent study showed that only about 5% of patients with primary prevention indications experience monomorphic VT episodes slower than 360 ms. [21] Setting a VT monitor zone between 360 and 330 ms is designed to reduce the inappropriate detection of SVTs, but still allow for identification of VT episodes below the therapy zone.

##### ○ longer detection duration

VF initial beats to detect is set to 30 of 40 beats which may reduce detection of fast but nonsustained SVTs.

##### ○ PR logic (dual chamber) or Wavelet (single chamber)

SVT discriminators reduce detection of sustained SVTs of 300 ms (SVT Limit) or slower.

### Statistical analysis

The primary endpoint is the difference in Morbidity Index incidence density between the study patients programmed to the PREPARE settings and the control population programmed to physician-tailored programming. The PREPARE programming strategy will be considered superior to the physician-tailored programming approach if a statistically significant reduction in the incidence density is demonstrated in the PREPARE group when compared to the historical control.

There were 347 shocked spontaneous VT/VF episodes, 4 events of arrhythmogenic syncope, and no episodes of untreated symptomatic sustained VT/VF in the 691 control population patients followed for 503 years (ID = 0.70). Assuming that the PREPARE programming strategy results in a 25% reduction in the number of shocked spontaneous VT/VF episodes, the rates of arrhythmogenic syncope and untreated symptomatic sustained VT/VF events remain the same, and the same number of years of follow-up, a total of 700 patients will provide at least 95% power for the primary hypothesis, tested at the two-sided significance level of 0.05. Both the Morbidity Index ID and the secondary analysis of the Morbidity Tachycardia Index ID will be tested using the comparison of incidence rates test. [22]

## Summary

The PREPARE study evaluates the ability of a pre-specified programming strategy to reduce VT/VF episode-related morbidity using a multi-center, prospective cohort design. The primary prevention population of 700 defibrillator patients with and without resynchronization therapy will be compared to 691 historical control patients. The VT/VF episode-related morbidity is assessed by a primary objective that indexes, for the number of years of follow-up, the total number of shocked spontaneous VT/VF episodes, untreated symptomatic sustained VT/VF episodes, and arrhythmogenic syncopal events.

The use of ICD and CRT+ICD devices in the primary prevention patient population has expanded dramatically over the last five years. Optimal programming for these patients has been investigated by other trials and understanding of these programming options continues to evolve. The programming choices available in the current devices enable additional specificity as it relates to the detection and treatment of ventricular arrhythmias. The PREPARE study results will increase the understanding of how to better develop programming strategies that positively impact primary prevention patients and their caregivers.

## Abbreviations

AF/Afl: Atrial Fibrillation/Atrial Flutter

ATP: Anti-tachycardia Pacing

bpm: beats per minute

CRT: Cardiac Resynchronization Therapy

CRT + ICD: Cardiac Resynchronization Therapy with Implantable Cardioverter Defibrillator

EGM: Electrogram

EMPIRIC Evaluation of EMPIRIC Programming to Improve Patient Management

FVT: Fast Ventricular Tachycardia

FVTDI Fast Ventricular Tachycardia Detection Interval

HR Heart rate

ICD: Implantable Cardioverter Defibrillator

ID Incidence Density

MIRACLE ICD: Multicenter InSync ICD Randomized Clinical Evaluation Trial

ms: milliseconds

PREPARE Primary Prevention Parameters Evaluation

Rx Therapy

ST/Sinus Tach: Sinus Tachycardia

SVT: Supraventricular Tachycardia

VF: Ventricular Fibrillation

VFDI: Ventricular Fibrillation Detection Interval

VF NID: Ventricular Fibrillation Number of Intervals to Detect

VF RNID: Ventricular Fibrillation Number of Intervals to Redetect

VT: Ventricular Tachycardia

VTDI: Ventricular Tachycardia Detection Interval

VT NID: Ventricular Tachycardia Number of Intervals to Detect

## Competing interests

1. In the past five years have you received reimbursements, fees, funding, or salary from an organization that may in any way gain or lose financially from the publication of this manuscript, either now or in the future?

Dr. Wilkoff: I am a consultant for Medtronic, St. Jude Medical and Guidant. Each of these companies manufactures ICDS.

Dr. Sterns: Medtronic Adverse Event Advisory Committee (AEAC), Medtronic

Lecturer

Dr. Williamson: Medtronic AEAC member

Dr. Wathen: Yes, Medtronic consultant

Holloman, Fieberg, Brown: Yes, salaried employees of Medtronic

2. Is such an organization financing this manuscript (including the article-processing charge)? If so, please specify.

Dr. Wilkoff, Dr. Stern, Dr. Williamson, Dr. Wathen: Holloman, Fieberg, Brown: Yes, Medtronic is paying for the processing fee of this article.

3. Do you hold any stocks or shares in an organization that may in any way gain or lose financially from the publication of this manuscript, either now or in the future? If so, please specify.

Dr. Wilkoff, Dr. Stern, Dr. Williamson, Dr. Wathen: No

Holloman, Fieberg, Brown: Yes, own Medtronic stock

4. Do you hold or are you currently applying for any patents relating to the content of the manuscript?

Dr. Wilkoff, Dr. Stern, Dr. Williamson, Dr. Wathen: Holloman, Fieberg, Brown: No

5. Have you received reimbursements, fees, funding, or salary from an organization that holds or has applied for patents relating to the content of the manuscript? If so, please specify.

Dr. Wilkoff, Dr. Stern, Dr. Williamson, Dr. Wathen: Holloman, Fieberg, Brown: No

6. Do you have any other financial competing interests? If so, please specify.

Dr. Wilkoff, Dr. Stern, Dr. Williamson, Dr. Wathen: Holloman, Fieberg, Brown: No

## Authors' contributions

BLW is Principle Investigator for the study described in the manuscript. BLW made significant contributions to study design, drafting, revising the manuscript, and provided final approval of version to be published. RS served on the AEAC for the study described in the manuscript, participated in revising the manuscript and review prior to final approval of version to be published. BW served on the AEAC for the study described in the manuscript and participated in revising the manuscript and review prior to final approval of version to be published. MW served on the AEAC for the study described in the manuscript, provided input into specific aspects of study design and participated in review of the manuscript and review prior to final approval of version to be published. KH participated in the design of the study described in the manuscript, acquisition, analysis and interpretation of data for the study, drafting and revising the manuscript, and participated in review of the final version to be published. AF performed statistical analysis, participated in modification to the study design, drafting and revising the manuscript, and participated in review of the final version to be published. MB participated in development of the study concept, revisions to the manuscript and review of the final version to be published.
